# Ultrashort Wave Combined with Human Umbilical Cord Mesenchymal Stem Cell (HUC-MSC) Transplantation Inhibits NLRP3 Inflammasome and Improves Spinal Cord Injury via MK2/TTP Signalling Pathway

**DOI:** 10.1155/2020/3021750

**Published:** 2020-12-05

**Authors:** Li Na, Shuai Wang, Tongtong Liu, Lixin Zhang

**Affiliations:** ^1^Department of Rehabilitation, Shengjing Hospital of China Medical University, Shenyang, Liaoning 110134, China; ^2^Department of Neurology, The People's Hospital of Liaoning Province, No. 33 Wenyi Road, Shenyang, Liaoning 110016, China; ^3^Department of Neurology, The First Affiliated Hospital of Sun Yat-sen University, Guangzhou, Guangdong 510080, China

## Abstract

**Objective:**

To investigate the curative effects of HUC-MSCs combined with USW on spinal cord injury (SCI) and the effects on inflammatory microenvironment and to explore the regulatory mechanisms of MK2/TTP signalling pathway and NLRP3 inflammasome.

**Methods:**

The SCI rat model was established using the modified Allen method; rats were administered with USW, HUC-MSCs, and combination therapy of USW and HUC-MSCs; the therapeutic efficacies in each group of rats were monitored and represented in BBB score. SCI levels were observed using HE staining and IF. The microglia polarisation state and released contents of inflammatory factors were detected. IF and Western Blotting were performed on to detect the expression levels of MK2/TTP signalling pathway and NLRP3 inflammasome-related proteins. Furthermore, the regulatory mechanisms of MK2/TTP pathway and NLRP3 were explored by performing on the *in vitro* study.

**Results:**

Combination therapy of USW and HUC-MSCs was found of significant efficacy on improving motor functions of SCI rats, and it was further proved that this combination therapy can reduce spinal cord injury in SCI rats, of which USW plays a more important role. While transplantation of HUC-MSCs can promote microglial cells developing to SCI repair, and M2 microglial cells were taking advantages gradually. The combination therapy can inhibit the expression of MK2; downregulate NLRP3 inflammasome; suppress the expression levels of pro-caspase-1, pro-IL-1*β*, and pro-IL-18; and simultaneously suppress the release of IL-1*β* and IL-18, thereby reducing spinal cord neurons apoptosis. It was found that the steady state of microglial polarisation maintained by combined treatment of USW and HUC-MSCs was destroyed with the upregulation of MK2 expression in cells, of which, M1 type microglial cell was dominant and the increased contents of inflammatory factors were detected. However, overexpressed MK2 relieved the inhibition of NLRP3 expression by TTP.

**Conclusions:**

Combination therapy of USW and HUC-MSCs can downregulate NLRP3 expression, relieve inflammatory responses, improve immune microenvironment, and rescue spinal cord injury via suppressing phosphorylation level of MK2.

## 1. Introduction

Spinal cord injury (SCI) usually leads to the patient's loss of feeling below the injury level and motor dysfunction, which greatly reduces the patient's quality of life [1]. It is a disease that seriously harms human health, and it was reported that its disability rate and mortality rate are extremely high [2]. SCI is generally caused by mechanical external forces, and the subsequent secondary injuries include inflammation, hypoxia, and demyelination,etc., forming a complex inflammatory microenvironment and causing nerve cell death, resulting in motor, sensory, and autonomic dysfunction [3–5]. Therefore, inhibiting inflammation and promoting spinal cord neuron regeneration are effective ways to treat SCI.

Studies have suggested that human umbilical cord mesenchymal stem cells (HUC-MSCs) used in the treatment of SCI rats can improve motor function, promote nerve growth factor expression, and inhibit IL-1*β* secretion [6–8]. The severe microenvironment of the injury site is a primary reason for limiting the clinical application of HUC-MSCs, as the occurrence of SCI can break the original balance of the spinal cord microenvironment, and the inflammatory cells and inflammatory factors in the microenvironment can cause the apoptosis of the implanted mesenchymal stem cells (MSCs), inhibiting the posttransplanted survival rate in the body [9, 10]. Moreover, *in vitro* studies have shown that inflammatory factors can affect the proliferation, migration, differentiation, and paracrine functions of MSCs [11]. IFN-*γ*, TNF-*α*, and IL-1*β* can inhibit MSC proliferation and affect the differentiation trend of MSCs [12], suggesting that the SCI-caused immune microenvironment has limited the survival rate and therapeutic effects of HUC-MSCs after transplantation.

As a physical therapy, low-dose ultrashort wave (USW) has achieved good results in the treatment of various acute inflammations, and it can also assist in inhibiting inflammation, eliminating oedema, improving blood circulation, and promoting nerve regeneration [13]. We have previously confirmed that USW can improve the immune microenvironment of SCI rats, reduce the expression of TNF-*α* and IL-1*β*, and improve the motor functions of rats. Its mechanism of action is related to inhibit the expression of MAPK-activated protein kinase 2 (MK2), which is involved in the regulation of cell cycle, cell migration, actin remodelling, and inflammation as a substrate of p38 MAPK. In inflammatory diseases, MK2 can activate the RNA binding protein tristetraprolin (TTP), a member of the zinc-finger protein family, thereby regulating the translation of TNF-*α* and inflammatory factors and promoting the relative expressions [14, 15]. Meanwhile, TTP is a negative regulator of the NLRP3 inflammasome, inhibiting the expression of NLRP3 through target binding to the 3′-UTR region of NLRP3, speculating that MK2 might release the inhibition of the NLRP3 expression by TTP and increase the NLRP3 expression through phosphorylating TTP [16]. NLRP3 inflammasome is the central process of inflammatory responses; its overactivation can continuously cleave pro-IL-1*β* and pro-IL-18 into mature forms by activated Caspase-1, thereby activating downstream signal transduction pathways, leading to the accumulated production of inflammatory mediators, causing severe inflammatory responses and promoting progression and development of multiple inflammatory diseases [17, 18]. In this study, depending on the improvement effects of USW on microenvironment, combination therapy with HUC-MSCs was used to treat SCI to observe the therapeutic efficacy and its regulatory effects on inflammatory microenvironment, in order to investigate the regulatory mechanisms of MK2/TTP signalling pathway and NLRP3 inflammasome, providing scientific evidences and theoretical basis on improving treatment with HUC-MSC transplantation and its clinical realisation.

## 2. Materials and Methods

### 2.1. Experimental Animals

Fifty SPF-grade SD male rats, weighting from 220 to 260 g, were purchased from Vital River Laboratory Animals Techniques (Beijing, China) (Production License No. SCXK (Beijing) 20120007). The animal experiments were carried out in barrier system of the Department of Laboratory Animal Sciences of China Medical University (User License No. SYXK (Liao) 2013001) and were approved by Laboratory Animal Welfare and Ethics Committee of China Medical University (IACUC No. 2019PS461K). Rats were randomly divided into the following five groups: sham operation group (Sham group, *n* = 10), SCI injury model group (SCI group, *n* = 10), SCI rats treated with USW group (USW group, *n* = 10), SCI rats treated with HUC-MSCs group (MSCs, *n* = 10), and SCI rats treated with combination therapy of USW and HUC-MSCs group (CT group, *n* = 10) [7]. The randomly method was performed as the random digital table method for randomly grouping.

### 2.2. Preparation and Identification of HUC-MSCs

Umbilical cords were collected, sterilised with 75% ethanol, and cleaned with PBS buffer. Amniotic membrane and blood vessels were peeled off, and the posttreated umbilical cords were cut into tissue sections with a diameter of 1-3 mm with a sterilised surgical scissors. Tissues sections were cultured in T75 culture flasks with 15 ml of culture medium DMEM (#11965092, Gibco, USA) containing 10% fetal bovine serum (FBS) (#16140071, Gibco, USA) and 1% poly-antibiotics of penicillin and streptomycin (P4333, Sigma-Aldrich, USA) in a preset cell culture incubator (37°C, 5% CO_2_). Culture medium was changed every four days. Tissue sections were removed when cell fusion rate in each group reached about 80-90%; cells were passaged at a ratio of 1 : 2 and collected on the 15th-20th day.

The identification of HUC-MSCs was performed using flow cytometry and immunofluorescence. Flow cytometry was used to detect the surface markers including CD90-FITC, CD77-FITC, CD105-FITC, CD34-phycoerythrin (PE), CD44-PE, CD29-FITC, CD14-FITC, HLA-DR, and CD45-PC7. HUC-MSCs cells grew to 80-90% confluence; cells were detached with 0.025% trypsin. Then, culture medium was added; after centrifugation, cells were diluted to 1 × 10^6^ cells/ml. Cell suspension was transferred, filtered, and packed into the sample tubes, each containing 200 *μ*l. Then, the mentioned antibody was added to sample tubes with incubation for 30 min at room temperature. Cells were washed and centrifuged by PBS and adjusted to 500 *μ*l. Then, the results were detected by flow cytometry.

The HUC-MSCs were seeded at 1 × 10^5^ cells/ml and 3 ml/well in a 6-well plate with a cover glass. After culture for 24 h at 37°C, cells were fixed with paraformaldehyde. Then, the cover glasses were rinsed by PBS for 3 times. Then, goat serum was dripped on the glass, and fibroblasts were sealed for 30 min at room temperature. After that, diluted CD105, CD29, CD44, CD73, and CD45 primary antibodies were dripped into each cover glass, incubating in a wet box at 4°C overnight. The second day, the secondary antibody goat anti-rat IgG H&L (1/200, ab150113) was added. Then, the glass was dripped with 4′,6-diamidino-2-phenylindole (DAPI) for nucleus staining, sealed with sealing liquid. And cells were observed under the fluorescence microscope.

### 2.3. SCI Model Establishment

The modified Allen method was used to establish the SCI rats model [19], and rats were anaesthetised with intraperitoneal injection of 1% pentobarbital sodium (P-010, Sigma-Aldrich, USA). A 2 cm long incision was made along the spine of rat at the central of T10, and the subcutaneous tendons were separated in order. Membranes and free sacral spinal muscles next to the spine were separated to reveal the spinous processes and vertebral bodies of the rat. According to the Allen's method, using a 10 g impact rod from 10.00 mm height, free fall to cause mild spinal cord injury, the strength of the strike (gram-force) was maintained at 125 g ·mm. After falling and impacting, retraction and flutter at lower limbs were obviously noticed, and the rat tail was spasmodically swayed, proving that the SCI rat modelling was successfully established. In the Sham group, the lamina was removed without spinal cord impacting, leading to no substantive spinal cord injury. The rats in the USW group were given low-dose ultrashort wave treatment once a day for 7 min after SCI modelling. Rats in the MSC group were given local injection of 10 *μ*l HUC-MSCs (1 × 10^5^/*μ*l) at injury site after SCI modelling surgery. For the combination therapy in the CT group of rats, 10 *μ*l HUC-MSC (1 × 10^5^/*μ*l) local injection at injury site was performed on SCI rats and followed with once-a-day USW treatment at 24 h post-injection and continuous for 14 days in total.

### 2.4. Basso, Beattie, and Bresnahan (BBB) Score

Hind limb motor function of each group of rats was evaluated at 1 d, 1 w, 2 w, and 4w after SCI modelling accessed by Bassobiti motor function evaluation scale [20] (Table 1). Briefly, rats were placed on the test platform to observe the lower limb joint activity, gait, stability of the trunk, fine movement of the paw, position of the tail, and coordination of the body. Scoring criteria were from minimum of 0 point (no visible hind limb movements, complete paralysis) to the maximum of 21 points (sustained palm movement, coordinated gait, toe grip, torso stability, tail lift, parallel of active claw position and body, completely normal movement). Rats' motor functions were evaluated and assigned into one of the following three categories, depending on their BBB grades.

### 2.5. Haematoxylin and Eosin (HE) Staining

Tissue sections fixed in formalin were collected, dehydrated with gradient ethanol (70%, 80%, 90%, and 100%), and transparentised with xylene. After that, tissue sections were made into paraffinized sections followed by being sliced into 4 *μ*m. Slices were then stained with haematoxylin and eosin staining solution and rinsed with running distilled water for the removal of residual staining solution. Slices were then embedded with neutral resin. Visualised observations were taken under a light microscope (CX33, Olympus Corporation, Japan).

### 2.6. Immunofluorescence (IF)

Deparaffinised spinal cord tissue sections were rehydrated and applied with 3% H_2_O_2_ solution and rinsed with PBS buffer. Antigen retrieval was done with applying 0.1 M sodium citrate solution. Then, sections were blocked with goat serum with a 30 min incubation at 37°C. Residual serum was removed by then. Primary antibodies against NeuN (#702022, Invitrogen, USA), iNOS (PA1-036, Invitrogen, USA), arg-1 (PA5-29645, Invitrogen, USA), IBA-1 (ab5076, Abcam, UK), NLRP3 (MA5-32255, Invitrogen, USA), and MK2 (ab131531, Abcam, UK) were applied to the sections followed by the overnight incubation at 4°C. After the removal of diluted primary antibody solutions, sections were rinsed with PBS buffer solution, ant incubated with fluorescent-labelled secondary antibody solution at 37°C for 30 min. DAPI staining solution was performed onto the sections for cell nucleus staining after thorough rinse with PBS buffer. Antifade reagent was applied to the sections after that to block the sections. Sections were then observed with a fluorescent microscope.

### 2.7. Terminal Deoxynucleotidyl Transferase dUTP Nick End Labelling (TUNEL)

Spinal cord neuron apoptosis was detected using an in situ cell apoptosis detection assay kit (C10618, Invitrogen, USA). Manufacturer's instruction was strictly followed in this experiment. Specifically, paraffin-embedded sections were deparaffinised with xylene (5 min/change, twice) and administered at 37°C for 30 min with 20 *μ*g/ml protease K excluding DNase. After that, sections were rinsed thoroughly with PBS buffer. TUNEL working solution was then added onto the sections in a volume of 50 *μ*l, followed by the incubation at 37°C for 1 h protected from light. DAPI staining solution was then performed onto the sections for cell nucleus staining with a 10-minute incubation at room temperature to avoid from light. Antifade working solution was finally added onto the sections after three changes of thorough rinse with PBS solution. Spinal cord cell apoptosis was detected using a fluorescent microscope.

### 2.8. Enzyme-Linked Immunosorbent Assay (ELISA)

Contents of M-CSF (SEA090Ra), IL-6 (SEA079Ra), IL-10 (SEA056Ra), IL-1*β* (SEA563Ra), IL-18(SEA064Ra) and TNF-*α* (SEA133Ra) in rats' spinal cords were determined using ELISA kits (Cloud-Clone Corp., USA). Protocols of each ELISA kit were followed in this experiment. Briefly, standard and diluted sample solutions were added into each well of ELISA plates in turn, and plates were incubated at 37°C for 1 h. Standards and samples were removed after incubation, and plates were washed with washing buffer solution provided in the ELISA kits. Detection reagents A and B were added into each well separately with the washing process performed on between adding the two detection reagents. TMB substrate was added after completely removing detection reagent B. Plates were incubated with TMB substrate at 37°C for 15-20 min protected from light. It was observed that the colour in each well turned into blue after incubation with TMB. With the addition of stop solution, colour changed into yellow directly, and the optical density values were determined within 10 min after colour changed using a microplate reader at 450 nm. Contents of each indicator were calculated using the obtained data and standard curves.

### 2.9. Western Blotting

Frozen spinal cord tissue sections stored in liquid nitrogen were taken out, and proteins were extracted with the addition of 1 ml RIPA lysis buffer (#89901, Thermo Scientific, USA) containing 2% protease inhibitor and 1% PMSF followed by completely homogenisation using an ultrasound homogeniser. Tissue homogenisations were then centrifuged at 12,000 rpm for 10 min, and the supernatants were collected and stored in -80°C for later use. Protein concentrations were quantified using BCA assay. Concentrated protein samples were then loaded into SDS-PAGE gels for electrophoresis and transferred onto 0.45 *μ*m PVDF membranes. Membranes were then incubated with diluted primary antibodies against MK2 (ab131531, Abcam, UK), p-MK2 (ab131504, Abcam, UK), TTP (ab124024, Abcam, UK), NLRP3 (ab263899, Abcam, UK), pro-caspase-1 (ab179515, Abcam, UK), pro-IL-1*β* (ab205924, Abcam, UK), iNOS (ab15323, Abcam, UK), arg-1 (ab272887, Abcam, UK), and GAPDH (ab8245, Abcam, UK) as loading internal control at 4°C for 12 h. On the next day, membranes were incubated with HRP-conjugated secondary antibody for 1 h at room temperature after rinse with TBST buffer solution for 3 changes. ECL chemo-reagents were then applied onto the membranes, and membranes were captured using an imaging system. Grey values of protein bands were detected and analysed using the ImageJ software.

### 2.10. Rat Spinal Cord Microglia Cultivation and Administration

Spinal cord microglia cells were isolated and cultivated into the following groups: control group (control group), microglia cells were cultured in DMEM medium containing 10% FBS; LPS induction group (LPS group), microglia cells were administered with 0.5 mg/L LPS for 24 h; LPS induction with the USW treatment group (USW + LPS group), LPS-induced microglia cells were treated with USW (service power 40 W, wavelength 7.37 m, practical output power 11 W) for 7 min; HUC-MSCs and LPS induction coculture microglia cell group (HUC − MSCs + LPS group), LPS-induced microglia cells were cocultured with HUC-MSCs; combination therapeutic treatment group (CT + LPS group), LPS-induced microglia cells were cocultured with HUC-MSCs and treated with 7-minute USW (service power 40 W, wavelength 7.37 m, practical output power 11 W); combination therapy treated LPS-induced MK2up microglia cells (CT + MK2up + LPS group), microglia cells were transfected with MK2-overexpressed viral vectors, induced with LPS and treated with combination therapy as previously.

### 2.11. Statistical Analysis

Obtained data were analysed using SPSS 21.0 Statistics (IBM SPSS Statistics, USA); data were represented in mean ± standard deviation (SD). One-way analysis of variance (ANOVA) was performed on the comparisons among multiple groups, least significant difference (LSD) test was used for comparisons between groups, and Wilcoxon rank-sum test was used when data were distributed nonnormally. Statistical significance was considered when *p* < 0.05.

## 3. Results

### 3.1. HUC-MSCs Cells Were Cultivated and Identified Successfully

The results of flow cytometry demonstrated that surface markers of HUC-MSCs including CD29, CD44, CD73, CD90, and CD105 were all positive, while the positive rates of CD14, CD34, HLA-DR, and CD45 were only 0.38%, 0.12%, 0.18%, and 0.12%, respectively (Figure S1A)). Immunofluorescence staining showed that HUC-MSCs were positive for the surface markers CD105, CD29, CD44, and CD73; however, they are negative for hematopoietic stem cell marker CD45 (Figure S1B). These results fulfilled the criteria of MSCs definition.

### 3.2. Combination Therapy Improves Motor Functions and Reduces Spinal Cord Injury in SCI Rats

**Figure 1:**
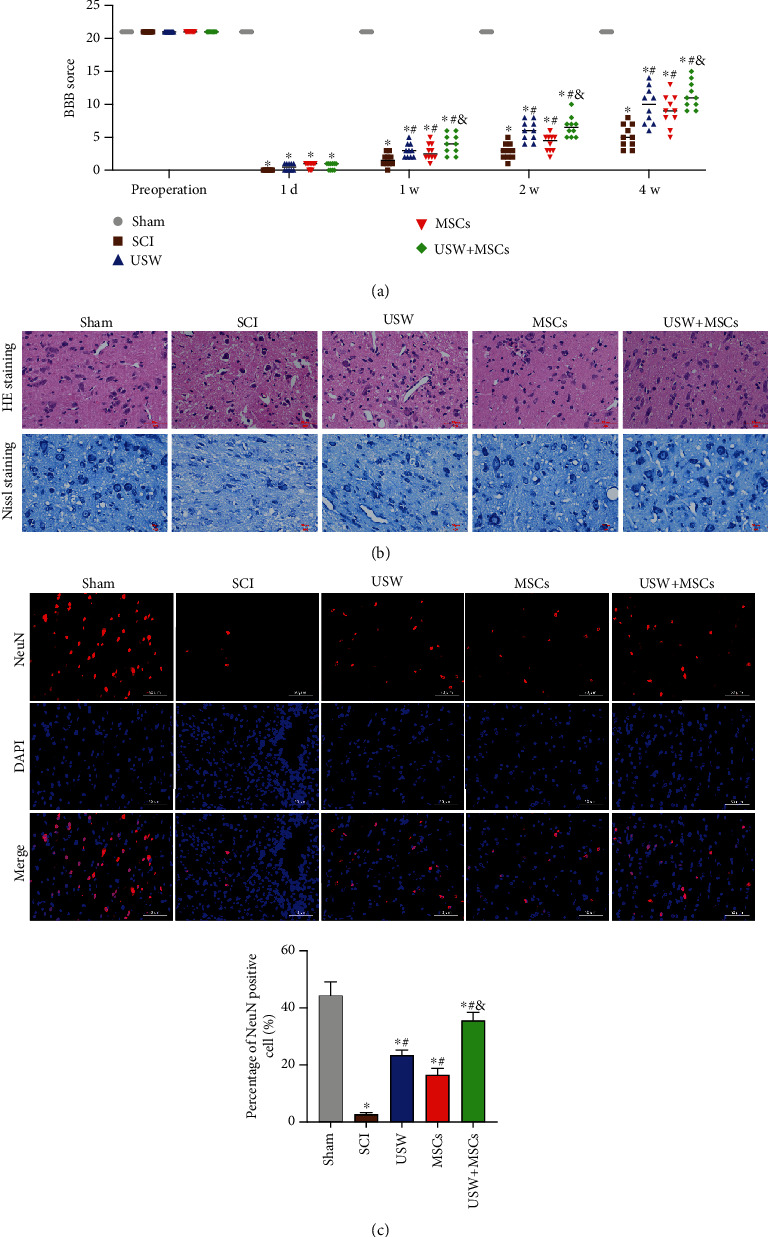
Combination therapy improves motor functions and reduces spinal cord injury in SCI rats. (a) Evaluated BBB scores in each group of rats in different time-points (preoperation, 1 d, 1 w, 2 w, and 4 w). (b) Histopathological changes in SCI rats treated with monotherapies of USW and HUC-MSCs, respectively, and combination therapy were observed with HE and Nissl staining (scale bar = 50 *μ*m). (c) Expression level of NeuN detected with IF (scale bar = 50 *μ*m); percentage of NeuN positive cells was calculated and represented in bar chart attached. “^∗^, #, &” indicated significant difference (*p* < 0.05), “^∗^” vs. Sham group, “#” vs. SCI group, and “&” vs. rats treated with monotherapy (USW/HUC-MSCs).

On the 1st day of SCI modelling in rats, it was found that rats completely lost motor functions in back limbs; BBB scores of all rats were evaluated at 0 score. BBB scores were gradually elevated at 2-4 w after SCI modelling, and BBB scores of rats in the USW and CT groups were significantly greater than that of rats in the SCI group. Interestingly, there was no significant difference between rats in the MSC and SCI group (Figure 1(a)), suggesting that USW treatment plays a more crucial role within the combination therapy of USW and HUC-MSCs. Histopathological results demonstrated that necrotic tissues and cell debris were found on the dorsal side of the spinal cords after SCI occurred, and inflammatory cell infiltration was obvious (Figure 1(b)). NeuN was detected using IF, illustrating that the number of neurons was decreased with SCI occurrence (Figure 1(c)), while monotherapy with USW and combination therapy were both found of improvement efficacy on SCI. In summary, USW treatment plays an essential role while assisted with MSC treatment; the above combination therapy can rescue spinal cord injury in SCI rats, suggesting that the relevant mechanism is correlated to the improvement of blood circulation, nutrition in tissues, and reduction of inflammatory oedema.

### 3.3. Combination Therapy Suppresses Inflammatory Responses and Improves Inflammatory Microenvironment in SCI Rats

**Figure 2:**
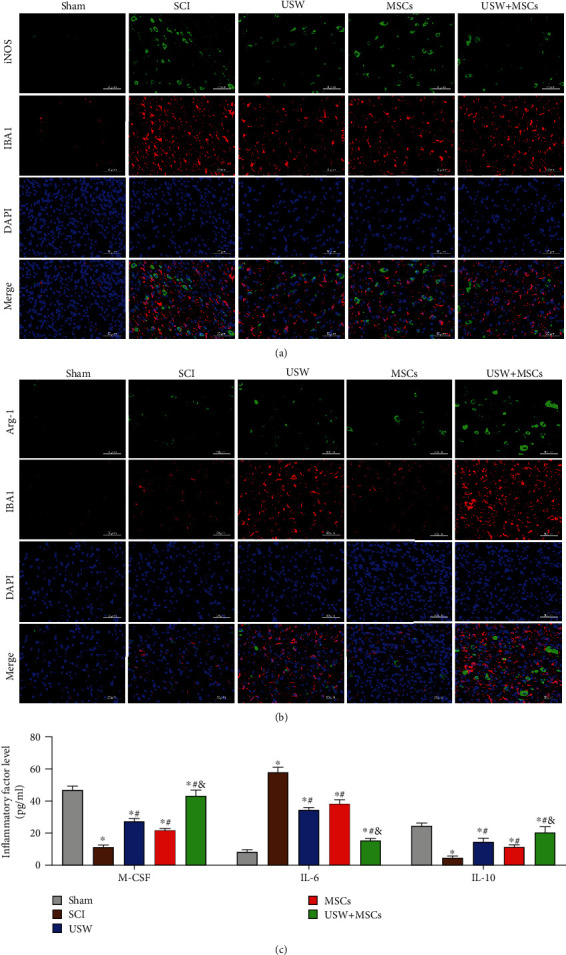
Combination therapy suppresses inflammatory responses and improves inflammatory microenvironment in SCI rats. Polarisation markers (iNOS and arg-1) of M1/M2 microglial polarisation states detected with IF represented in iNOS/IBA-1 (a) and arg-1/IBA-1 (b) costaining (scale bar = 50 *μ*m). (c) Contents of inflammatory factors (M-CSF, IL-6, and IL-10) determined using ELISA. “^∗^, #, &” indicated significant difference (*p* < 0.05), “^∗^” vs. Sham group, “#” vs. SCI group, and “&” vs. rats treated with monotherapy (USW/HUC-MSCs).

It was reported that SCI can lead to the activation of microglia cells and infiltration of macrophages, resulting in local inflammatory microenvironment. Microglia cells are polarised into M1 and M2 types from resting state after SCI occurrence. In this study, microglia cells were labelled using IBA-1, according to the observations of dual-labels of M1 type charactering factor iNOS and M2 charactering factor arg-1; it was found that microglia cells at rest state (M0) were polarised into M1 and M2 types, resulting in the elevated expression levels of iNOS and arg-1 (Figure 2(a)), and it was also determined that the overactivated M1 microglia cells secreted enormous inflammatory factors showing as the reduction of M-CSF and IL-10 and elevated level of IL-6 (Figure 2(b)). Accumulated M2 type microglial cells were found in the three treatment groups, resulting in the increasing level of arg-1, M-CSF, and IL-10, while a decreasing level of iNOS and IL-6(Figure 2(c)). Among the three treatment groups, the relevant observations were most significant in CT group, and no obvious differences were detected between USW and MSCs groups, suggesting that combination therapy can provide neurons with a more suitable microenvironment.

### 3.4. Combination Therapy Downregulates NLRP3 and Inhibits Microglia Pyroptosis in SCI Rats

**Figure 3:**
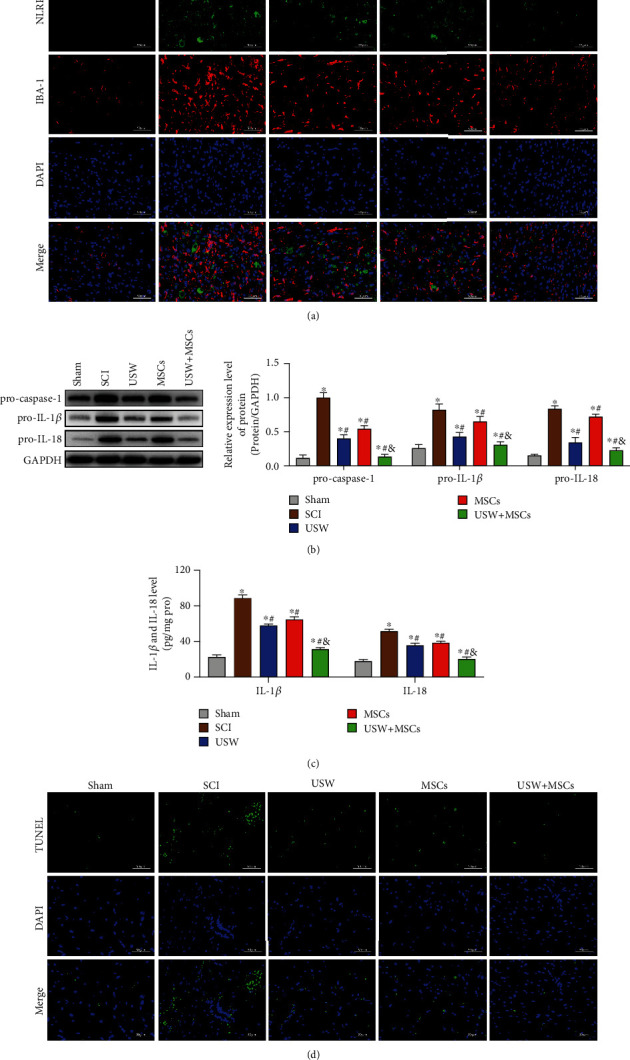
Combination therapy downregulates NLRP3 and inhibits microglia pyroptosis in SCI rats: (a) NLRP3/IBA-1 costaining detected by IF (scale bar = 50 *μ*m); (b) protein expression levels of pro-caspase-1, pro-IL-1*β*, and pro-IL-18 determined using Western Blotting; (c) levels of IL-1*β* and IL-18 determined using ELISA; (d) neuron cell apoptosis detected using TUNEL (scale bar = 50 *μ*m). “^∗^, #, &” indicated significant difference (*p* < 0.05), “^∗^” vs. Sham group, “#” vs. SCI group, and “&” vs. rats treated with monotherapy (USW/HUC-MSCs).

The ignition of inflammatory responses requires the involvement of protein complex of inflammasomes. It has been proved that the expression level of NLRP3 inflammasome was elevated after SCI occurs, inhibiting NLRP3 inflammasome can improve motor dysfunctions caused by SCI. In this study, the expression level of inflammasome in microglia cells was observed by dual-labelling IF; results showed that expression levels of IBA-1 and NLRP3 were both elevated in SCI rats (Figure 3(a)), and the increased level of NLRP3 further catalysed accumulated expression of pro-caspase-1, thereby promoting expressions of pro-IL-1*β* and pro-IL-18 (Figure 3(b)) and simultaneously matured into IL-1*β* and IL-18 with biological activity (Figure 3(c)) which were involved in the following inflammatory responses and resulted in the neuron cells apoptosis in spinal cords (Figure 3(d)). NLRP3 inhibition was detected in the three treatment groups, of which, monotherapy of HUC-MSCs exhibited a weaker inhibiting effect on inflammatory responses in microenvironment. However, combination therapy significantly inhibits pro-caspase-1, pro-IL-1*β*, and pro-IL-18 and simultaneously suppressed the release of mature IL-1*β* and IL-18, reduce apoptosis rate of spinal cord neurons, suggesting that the combination therapy of USW and HUC-MSCs can suppress inflammasome and reduce the SCI.

### 3.5. Combination Therapy Inhibits MK2 Phosphorylation in SCI Rats

**Figure 4:**
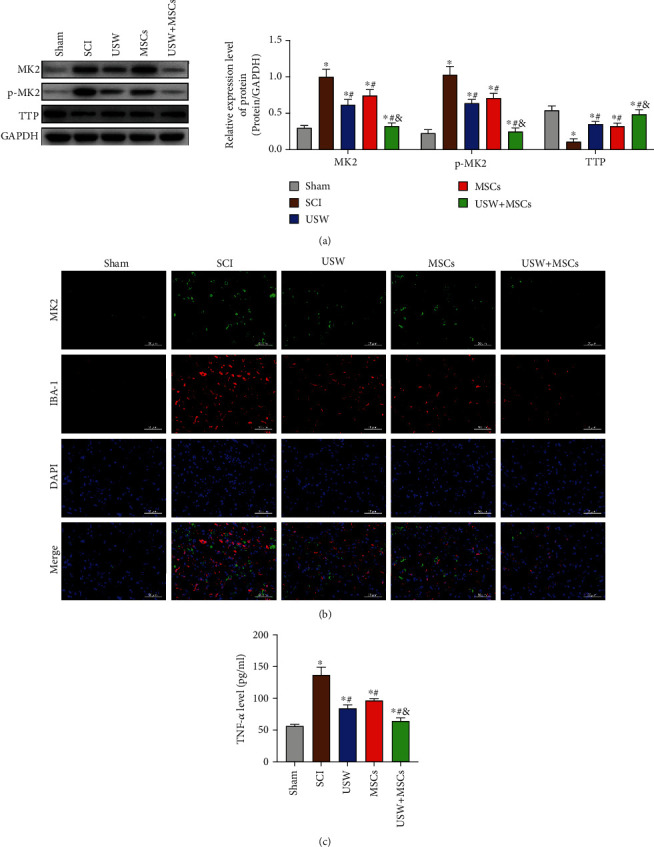
Combination therapy inhibits MK2 phosphorylation in SCI rats: (a) protein expressions and MK2 phosphorylation level detected using Western Blotting; (b) MK2/IBA-1 costaining detected using IF (scale bar = 50 *μ*m); (c) TNF-*α* level in microglial cells determined using ELISA. “^∗^, #, &” indicated significant difference (*p* < 0.05), “^∗^” vs. Sham group, “#” vs. SCI group, and “&” vs. rats treated with monotherapy (USW/HUC-MSCs).

We have previously determined that contents of MK2 were increased in patients' plasma, speculating that the increasing level of the MK2 gene expression might be related to the occurrence of local inflammatory microenvironment. In this study, we further investigated the effects of combination therapy on MK2 gene in SCI rats. Results in Figure 4 demonstrated that SCI can phosphorylate MK2 in rats, suppressed the activity of TTP (Figure 4(a)), thereby inducing the release of downstream factor, TNF-*α* (Figure 4(c)). Interestingly, the high level of MK2 was also detected in microglia cells (Figure 4(b)), suggesting that MK2 is involved in the inflammatory responses in SCI rats' microenvironment. MK2 gene was inhibited in different scales in the three treatment groups (monotherapy of USW or HUC-MSCs and polytherapy), of which, combination therapy exhibited the most significant therapeutic efficacy. With the combination therapy treatment, phosphorylation level of MK2 was inhibited, TTP expression was raised, and the content of TNF-*α* was reduced. It was hypothesised that SCI occurrence upregulated the MK2 expression, suppressed the TTP activity, promoted the NLRP3 expression, and increased the inflammatory factors excretion, thereby promoting the formation of local inflammatory microenvironment.

### 3.6. MK2 Upregulation Blocks the Microglia Polarisation Promoted by Combination Therapy

**Figure 5:**
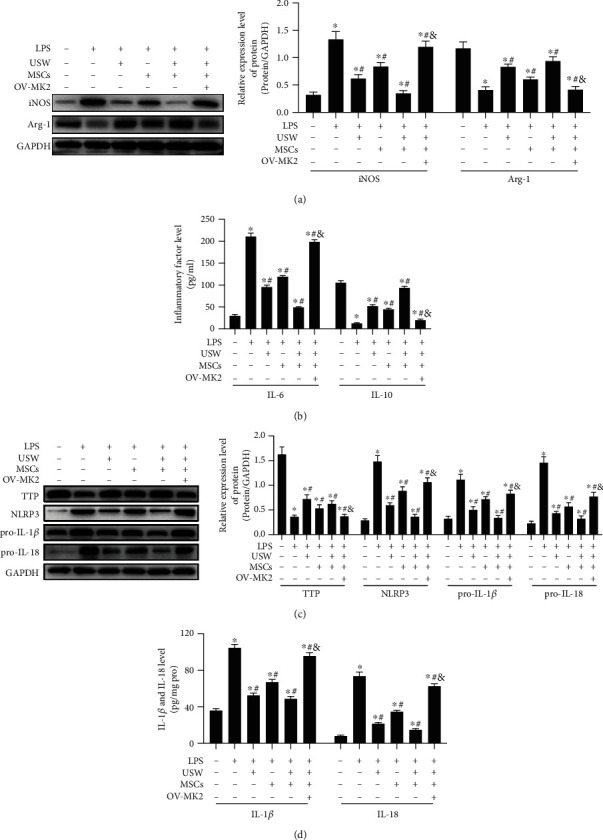
MK2 upregulation blocks the microglia polarisation promoted by combination therapy: (a) iNOS and arg-1 expression levels detected using Western Blotting; (b) contents of inflammatory factors (IL-6 and IL-10) determined using ELISA; (c) Western Blotting was performed on to determine the expression levels of TTP, NLRP3, pro-IL-1*β*, and pro-IL-18; (d) contents of IL-1*β* and IL-18 in cellular supernatant were quantified with ELISA. “^∗^, #, &” indicated significant difference (*p* < 0.05), “^∗^” vs. Sham group, “#” vs. SCI group, and “&” vs. rats treated with monotherapies and combination therapy.

To further explore the regulatory mechanisms of reducing spinal cord injury in SCI rats by combination therapy via MK2/TTP pathway and NLRP3, we cultivated rats' spinal cord microglia cells, established *in vitro* SCI model using LPS, treating with monotherapy of USW or cocultivation with HUC-MSCs and combination therapy. Results demonstrated that the combination therapy can regulate microglia M1/M2 polarisation state, suppress M1 polarisation, downregulate the iNOS expression, and elevate the arg-1 expression (Figure 5(a)). Meanwhile, it was detected that content of IL-6 was reduced and IL-10 level was raised with the treatment of combination therapy (Figure 5(b)). The microglia polarisation state was destroyed when MK2 gene was upregulated in cells, leading to the cells polarising to M1 state, resulting in the increasing levels of inflammatory factors. The over-expressed MK2 unlocked the inhibiting mechanisms of TTP on NLRP3 expression, thereby increasing the expression levels of NLRP3, pro-IL-1*β*, and pro-IL-18 (Figure 5(c)) and contents of IL-1*β* and IL-18 in cellular supernatants (Figure 5(d)), suggesting that combination therapy can downregulate the NLRP3 expression level through inhibiting phosphorylation of MK2, leading to the reduction of inflammatory responses, improving immune microenvironment, and rescuing SCI.

## 4. Discussion

SCI is a serious traumatic disease of central nervous system; the treatment and functional recovery exercise after SCI are still the focus and difficulty of clinical research at the present [21]. In this study, combination therapy of USW treatment and HUC-MSC transplantation was used to treat SCI; it was observed that different scale of recovery was detected from the first week, and it reached the most clinical significance at 4 w. From the results above, it can be confirmed that USW treatment plays a more important role in improving rats' motor dysfunctions, while HUC-MSC transplantation therapy plays as an assistant role. Combination therapy has been confirmed of a certain promoting effect on rescuing SCI due to the abilities of antiapoptosis, antioxidation, and anti-inflammation and promoting the regeneration of endogenous stem cells. Its relative regulatory mechanisms are related to the increasing level of nutrient functions in corresponding tissues, reducing inflammation-induced oedema, and improving the blood circulation in tissues.

The occurrence of SCI can break the balance state of the original microenvironment in spinal cords [22]. Damaged neurons, astrocytes, and other injured cells can secrete enormous inflammatory factors (IL-1*β*, IL-6, TNF-*α*, and IFN-*γ*), causing microglia activation and macrophages infiltration, forming local inflammatory microenvironment, leading to the extensive demyelination of living axons and inhibition of axon regeneration, thereby further blocking the regenerations of neuro-tissue structure and functions [23–26]. Microglia cells are in the classic dynamic balance of proinflammatory (M1 type) and anti-inflammatory (M2 type), and its transformation is related to the local microenvironment they are in [27, 28]. The occurrence of neuro-inflammatory responses can be possibly avoided by adjusting the polarisation balance of M1/M2 state, providing important significance protecting nervous system from secondary damage and important clinical evidences in the diagnosis and treatment of SCI [29, 30]. Both the monotherapy of USW treatment and HUC-MSC transplantation can reduce the activations of microglia and macrophages, decreasing inflammatory responses and improving local microenvironment at injury tissues. Notably, the combination therapy can lead to more effective therapeutic efficacy compared to each monotherapy. It has been reported that HUC-MSC transplantation can promote the development of microglial cells to SCI repair, showing the roles of immune regulation, tissue repair, and functional remodelling. While the USW treatment can enhance the immune functions, elevating the phagocytic ability of macrophages, improving blood circulation and tissue nutrition, promoting the discharge of inflammatory productions ,and is conductive to inflammation control and dissipation.

Inflammasomes are the important inflammatory factors involved in the inflammatory responses discovered in recent years; classical inflammasome pathways are mediated by Caspase-1, where the activated Caspase-1 can induce inflammatory responses and the classical pyroptosis also occurs in this way [18]. The activation of inflammasomes and its induced expressions of IL-1*β* and IL-18 are all detected in SCI tissues. Accumulated evidences reported that inhibiting NLRP3 inflammasome can activate and regulate nervous inflammation by drug administration to recover the SCI-caused nervous damage, indicating that NLRP3 inflammasome is the key factor regulating SCI-induced secondary injury in mice [31, 32]. In this study, we proved that the NLRP3 expression in SCI rats was considerably downregulated with combination therapy; meanwhile, Caspase-1 activation was inhibited, leading to the reduced expression levels of pro-IL-1*β* and pro-IL-18, thereby relieving inflammatory responses, decreasing cell apoptosis rate, and suppressing the secretions of matured IL-1*β* and IL-18, reporting that the combination therapy can reduce SCI via suppressing inflammasomes.

In our previous study, we have reported that the mRNA and protein expression levels of MK2 in SCI patients' plasma were both higher than those of volunteers without SCI disease, and it was also suggested that MK2 has regulatory effects on biosynthesis of TNF and other inflammatory factors, speculating that the regulatory effects might be related to the formation of inflammatory microenvironment [33]. Literatures indicated that less accumulation of TNF-*α*, IL-6, and macrophage inflammatory protein-2 (MIP-2) were detected in mice with MK2 knockout, showing a weaker inflammatory response. Other studies reported that levels of IL-1*β*, IL-18, and TNF-*α* were reduced through inhibiting inflammasome in colorectal carcinoma mice with inhibited MK2, leading to the reduction of inflammatory responses and suppression of tumour growth, suggesting that MK2 can promote the expression of NLRP3 inflammasome through activating TTP, thereby promoting inflammatory responses [34]. To further investigate the effective mechanisms of MK2/TTP pathway and NLRP3 in combination therapy-relieved SCI in rats, we primarily confirmed that the combination therapy inhibited MK2 phosphorylation in SCI rats, promoted the TTP expression, and suppressed the NLRP3 expression and inflammatory responses. Furthermore, the *in vitro* experiment was carried out in this study; the MK2 expression was upregulated in the SCI *in vitro* model. Results showed that the polarisation state regulated by the combination therapy was destroyed, and it was confirmed that M1 type primarily regulated the above mechanism. After the balance was destroyed, increasing level of inflammatory factors promoted cell apoptosis; meanwhile, the overexpressed MK2 unlocked the inhibiting effects on NLRP3 by TTP, indicating that combination therapy can downregulate the NLRP3 expression level via suppressing MK2 phosphorylation, thereby reducing inflammatory responses, improving immune microenvironment, and relieving SCI.

## Figures and Tables

**Table 1:** BBB score evaluation scale.

Score	Description	Stage of recovery
0-7	No visible hind limb movements, complete paralysis, or inaccessible joint movements	Early stage
8-13	Uncoordinated joint movement	Intermediate stage
14-21	Sustained movement, coordinated limb and joint movement, complete normal movement	Late stage

## Data Availability

The datasets used and/or analysed during the current study are available from the corresponding author on reasonable request.

## Supplementary Materials

Supplementary Materials Figure S1: the identification of hUC-MSCs. (A) Surface marker profiles of CD29, CD44, CD73, CD90, CD105, CD14, CD34, HLA-DR, and CD45 in hUC-MSCs were detected by flow cytometry. (B) Surface marker profiles of CD105, CD29, CD73, and CD45 were examined by immunofluorescence.
