# Supplementary material for: Ultrashort Wave Combined with Human Umbilical Cord Mesenchymal Stem Cell (HUC-MSC) Transplantation Inhibits NLRP3 Inflammasome and Improves Spinal Cord Injury via MK2/TTP Signalling Pathway
Source: Biomed Res Int. 2020 Dec 5;2020:3021750. doi: 10.1155/2020/3021750 (PMC7738785; doi:10.1155/2020/3021750)

**Supplementary materials:**

**Figure.S1 The identification of hUC-MSCs.**(A) Surface marker profiles of CD29, CD44, CD73, CD90, CD105, CD14, CD34, HLA-DR and CD45 in hUC-MSCs were detected by flow cytometry; (B) Surface marker profiles of CD105, CD29, CD73 and CD45 were examined by immunofluorescence.


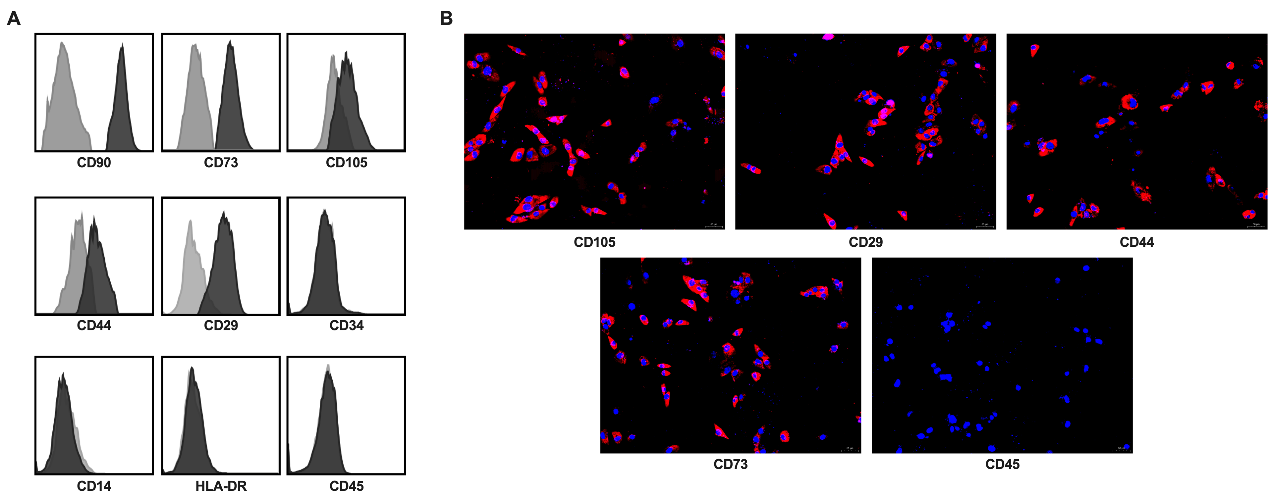

Supplement: Supplementary Materials — Figure S1: the identification of hUC-MSCs. (A) Surface marker profiles of CD29, CD44, CD73, CD90, CD105, CD14, CD34, HLA-DR, and CD45 in hUC-MSCs were detected by flow cytometry. (B) Surface marker profiles of CD105, CD29, CD73, and CD45 were examined by immunofluorescence. [file 3021750.f1.docx]
